# The effect of ceramic thickness on opalescence

**DOI:** 10.1002/cre2.325

**Published:** 2020-09-09

**Authors:** Sara Valizadeh, Alireza Mahmoudi Nahavandi, Marzieh Daryadar, Mutlu Özcan, Sedighe Sadat Hashemikamangar

**Affiliations:** ^1^ Dental Research Center, Dentistry Research Institute, Restorative Dentistry Department, School of Dentistry Tehran University of Medical Sciences Tehran Iran; ^2^ Color Imaging and Color Image Processing Department Institute for Color Science and Technology (ICST) Tehran Iran; ^3^ Restorative Dentist Tehran University of Medical Sciences Tehran Iran; ^4^ Division of Dental Biomaterials, Center for Dental and Oral Medicine, Clinic for Reconstructive Dentistry University of Zürich Zurich Switzerland; ^5^ Dental Research Center, Dentistry Research Institute, Department of Operative Dentistry, Dental School Tehran University of Medical Sciences Tehran Iran

**Keywords:** dental ceramics, Enamic, IPS e.max, opalescence, thickness, zirconia

## Abstract

**Objectives:**

Creating a tooth‐like appearance by use of dental ceramics is still a challenge. Opalescence is a unique property of dental enamel, attempted to be mimicked by dental restorative materials. This study aimed to assess the effect of ceramic thickness on opalescence.

**Materials and methods:**

Twenty‐four discs were fabricated of feldspathic ceramic, IPS e.max, zirconia and Enamic ceramics with 10 mm diameter and 0.5 and 1 mm thicknesses (n = 12). The opalescence of ceramic specimens was calculated by measuring the difference in yellow‐blue axis (CIE ∆b*) and red‐green axis (CIE ∆a*) between the transmitted and reflected spectra. One‐way ANOVA was applied to compare the opalescence of different ceramic specimens with variable thicknesses at .05 level of significance.

**Results:**

The opalescence of feldspathic, IPS e.max, zirconia and Enamic ceramic specimens with 0.5 mm thickness was 1.06 ± 0.15, 3.39 ± 0.15, 1.98 ± 0.15 and 1.44 ± 0.15, respectively. By increasing the thickness to 1 mm, the opalescence of feldspathic, IPS e.max, zirconia and Enamic ceramics changed to 1.12 ± 0.15, 1.47 ± 0.15, 3.85 ± 0.15 and 2.00 ± 0.15, respectively. In all groups except for IPS e.max, the mean opalescence of 1‐mm‐thick specimens was higher than that of 0.5‐mm‐thick specimens.

**Conclusion:**

Type and thickness of ceramic affect its opalescence. The opalescence of all ceramic specimens tested in this study with 0.5 and 1 mm thicknesses was lower than that of the enamel.

## 1 INTRODUCTION

Creating a natural tooth appearance with ceramic restorations is a challenge in cosmetic dentistry. In this respect, it is highly important to match the optical properties of these restorations with those of natural teeth. Factors such as the opalescence, fluorescence, translucency, surface properties, thickness and contour of restorations, ceramic brand and number of firing cycles can affect the final color of ceramic restorations (Begum, Chheda, Shruthi, & Sonika, 2014).

Tooth color is affected by factors such as the spectral distribution of the environmental light, sensitivity of the eye of the observer, and absorption, reflection and transmission of light; all these factors ultimately determine the final tooth color (Pires, Novais, Araújo, & Pegoraro, 2017).

Of all the optical properties required for an ideal dental restoration, the opalescence and fluorescence of restorative materials are highly important in addition to their value, hue and chroma (Monteiro, Brito, Pereira, & Alves, 2012). The human enamel is opalescent, and light scattering in wavelengths shorter than the visible spectrum confers a blue tint to the tooth color under reflected light and an orange‐brown tint under transmitted light (Cho, Yu, & Lee, 2009). The opalescence value ranges from 7.6 to 22.7 for the bovine enamel and 19.8–27.6 for the human enamel. Due to the opalescence of the human enamel, ideal ceramic restorations should have an opalescence similar to that of natural teeth (Lee & Yu, 2007).

The final color of ceramic restorations depends on their opacity and thickness as well as the color of the underlying tooth structure and luting cement. Moreover, the chemical composition of ceramic, the size of ceramic crystals and their inherent optical properties such as opalescence, fluorescence and translucency have a significant effect on the final color of restorations (Raptis, Michalakis, & Hirayama, 2006). When the refractive index between the two substrates (the ratio of higher to lower refractive index) is larger than 1.1, the object can have opalescence (Egen et al., 2004). The opalescence of dental materials is determined by the opalescence parameter, which is the difference in yellow‐blue axis (CIE ∆b*) and the red‐green axis (CIE ∆a*) between the transmitted and reflected light (Lee, 2016).

The opalescence and fluorescence of dental ceramics are evaluated to simplify the layering technique during their application. It has been generally accepted that opalescent esthetic restorations have improved masking ability. Opalescence and translucency of ceramics, if being in the same range, can play a role in masking of the underlying color. The effect of opalescence and fluorescence on light transmission of ceramics has also been studied as a function of light wavelength (Lee, Lu, & Powers, 2006).

Presence of micro‐particles or a glass phase in opalescent ceramics results in light scattering and eliminates many esthetic problems. It can also enable the simulation of translucency and opalescence of natural teeth (M Primus, Chu, Shelby, Buldrini, & Heckle, 2002).

All‐ceramic restorations can mimic the properties of natural teeth in terms of color and translucency. These restorations are often fabricated with different contours and thicknesses depending on intraoral conditions (Rosensteil, Land, & Fujimoto, 2014). An ideal color match is often difficult to achieve in the clinical setting even when the restoration is fabricated with adequate thickness. This is because of the wide range of translucency and opalescence of different ceramic types (Alp, Subaşı, Seghi, Johnston, & Yilmaz, 2018).

Type and thickness of ceramic materials are among the parameters that significantly affect the optical properties of restorations such as their opalescence. By a change in restoration thickness, its color, translucency and opalescence are also expected to change. In ceramic restoration of teeth, different ceramic thicknesses may be required depending on the type of restoration (Subaşı, Alp, Johnston, & Yilmaz, 2018; Tabatabaei, Nahavandi, Khorshidi, & Hashemikamangar, 2019).

The optical properties of ceramic restorations may be influenced by the color of the underlying substrate and luting cement. These properties can affect the color match of restorations depending on the ceramic thickness (Sari et al., 2018). Thus, it is imperative to assess the correlation of opalescence and ceramic thickness in different esthetic restorations to improve the clinical results. Although the effect of ceramic thickness on optical properties has been previously studied, comprehensive information about the effect of ceramic thickness on opalescence is still lacking. Thus, this study sought to assess the effect of thickness of different ceramic types on their opalescence.

## 2 MATERIALS AND METHODS

This in vitro, experimental study evaluated the following ceramic types due to their different composition: VM®9 feldspathic ceramic (A2 shade; Vita), IPS e.max (A2 shade, HT, Ivoclar), zirconia ceramic (A2 shade, Kerox^tm^ Zircostar), and Enamic® hybrid ceramic (A2 shade, Vita). Table 1 presents the characteristics of the materials used in this study.

**TABLE 1:** Composition of dental ceramics evaluated in this study

Ceramic type	Composition
Feldspathic ceramic	Metal, 15–25% quartz, Leucite, Potassium feldspar, NAlSi_3_O_3_, KALSi_3_O_3_ pigments, oxides
IPS e.max	SiO_2_, Li_2_O, K_2_O, P_2_O_5_, ZrO_2_, ZnO
Zirconia	ZrO_2_, Y_2_O_3_, Al_2_O_3_, SiO_2_, Fe_2_O_3_, Na_2_O
Enamic	SiO_2_, Al_2_O_3_, Na_2_O, K_2_O, B_2_O_3_, CaO, TiO_2_, PMMA

### 2.1 Preparation of specimens

A total of 24 discs were fabricated from each ceramic type; of which, 12 measured 10 × 0.5 mm and the remaining 12 measured 10 × 1 mm.

#### 2.1.1 Feldspathic ceramic

For the purpose of standardization of the size of specimens, cylindrical silicon molds with 0.5 and 1 mm depths and 10 mm diameter were used. The porcelain powder was mixed with distilled water according to the manufacturer's instructions and poured into the molds. Excess water was removed by vibration. The porcelain was condensed in the mold and baked after removal from the silicon mold according to the manufacturer's instructions. The baking process included heating at 450–919°C under 80 mbar vacuum. The temperature increased at a rate of 55°C/min to a maximum of 920°C. The ceramic remained at 920°C for 90 s.

### 2.2 IPS e.max

The wax pattern was designed in the desired dimensions using the computer‐aided design (CAD) system and milled in a milling machine. The discs with the desired dimensions were fabricated as such. Next, the wax patterns were sprued and flasked using a 100‐g flask, which was heated to 700°C in a furnace to eliminate the wax pattern. Next, the ceramic ingots were placed in the furnace. After heating to 910°C under vacuum, the ingot was injected into the sprue by the plunger. After condensation and cooling, the gypsum particles were removed using 100 μm aluminum oxide particles with 2.5 bar pressure. The specimens were then placed in Invex liquid and then in an ultrasonic bath for 4 min. They were then rinsed and dried. The impurities were removed from the surface of specimens using 100 μm aluminum oxide particles with 2.5 bar pressure. The sprues were cut by a wet diamond disc. The specimens were finally baked in a furnace with the following protocol: heating at 410–725°C under 80 mbar vacuum. The temperature increased at a rate of 60°C/min to a maximum of 730°C. The ceramic remained at 730°C for 60 s.

### 2.3 Zirconia

Ceramic specimens with the desired dimensions were first designed by the CAD system. Next, the zirconia blank was placed in the milling machine, and the ceramic discs with the desired dimensions were milled. The models were then separated from the blank by a diamond disc. Dust was removed from the specimen surface by air spray and the samples were placed on a plate for baking in a furnace. They were then baked at 1500°C for 120 min.

### 2.4 Enamic

Ceramic specimens with the desired dimensions were designed by the CAD system. The blocks were placed in a milling machine and the discs with the desired dimensions were fabricated as such. The dimensions of all specimens were measured by a caliper to ensure the desired thickness.

### 2.5 Assessment of color and opalescence

The reflection and transmission spectra were measured by CS 2000 spectrophotometer (Konika Minolta). The color coordinates of the specimens were calculated by the spectrophotometer software (CS10‐W) in CIEL*a*b* color space under D65/2^0^ observation conditions. For measurement of opalescence in the reflectance mode, the specimens were fixed on a jig and a white tile was placed in front of them. The device was calibrated with the white tile, and the reflectance spectrum of the specimen was measured. In the transmittance mode, the device was calibrated by a lamp light. The specimen was placed in front of the lamp light and the energy received by the device from the specimen was measured. The ratio of the reflectance and transmittance spectra was then calculated. Measurements were repeated twice and the mean values were used for statistcial analysis.

The opalescence was calculated using the formula below where the T and R indicate the transmittance and the reflectance modes, respectively.

$$\mathrm{OP}={\left[{\left(\mathrm{CIE}\;{a_{T}}^{*}-\mathrm{CIE}\;{a_{R}}^{*}\right)}^{2}+{\left(\mathrm{CIE}\;{b_{T}}^{*}-\mathrm{CIE}\;{b_{R}}^{*}\right)}^{2}\right]}^{1/2}$$

**FIGURE 1:**
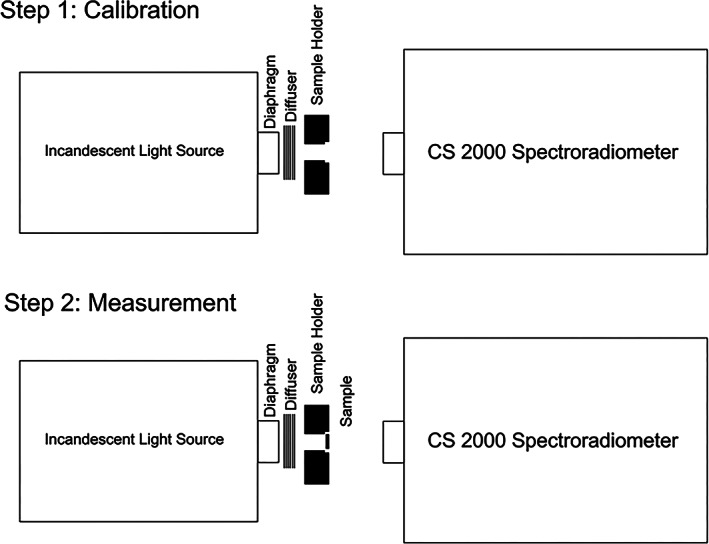
Measuring the transmittance

**FIGURE 2:**
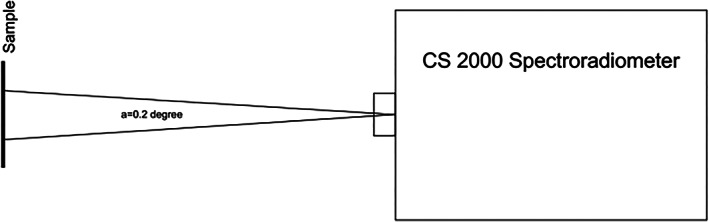
Measuring the color parameters at the center of specimen

A spectroradiometer (CS‐2000; Konika Minolta) was used to measure the transmittance and reflectance of the specimens. For measurement of transmittance, an incandescent light source was used powered by a constant power supply. A paper was folded in front of the power supply to obtain ideal emission of light. Next, a black plexiglass holder fabricated by a laser cutting machine was used to hold the specimens. Figure 1 illustrates the measurement of transmittance by a spectroradiometer. The transmittance was read with the angle of device adjusted at 0.2°. Considering 80 cm distance of the specimen from the spectroradiometer, a circle with 2.8 mm diameter at the center of the sample was measured (Figure 2).

**FIGURE 3:**
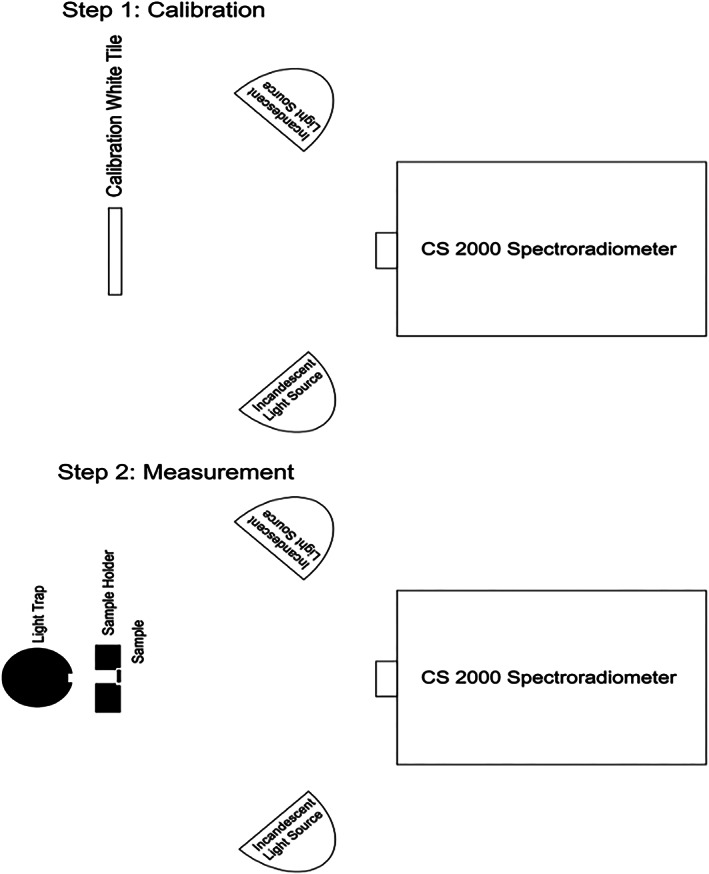
Measuring the reflectance

To measure the reflectance, two incandescent light sources illuminated the sample with 45° angle. The lamps were lit by a power source and the device was calibrated using a white tile. Next, the calibration white tile was removed and the specimens were placed at the site of the tile with a holder. Since the specimens were semi‐transparent, an optical trap was placed behind them to prevent the return of light reflection after passing through the specimen. The reflectance of the specimen was then read. Figure 3 illustrates the measurement of reflectance using a spectroradiometer.

The opalescence of the specimens was calculated using the difference in chromaticity of the specimens in transmittance and reflectance modes with the formula below:

$$\mathrm{op}={\left({\left(\Delta a^{*}\right)}^{2}+{\left(\Delta b^{*}\right)}^{2}\right)}^{0.5}$$

### 2.6 Statistical analysis

Data were analyzed using SPSS version 21. Two‐way ANOVA was used to compare the groups. According to Table 2, the effect of ceramic type on opalescence was significant (*p* = .000) while the effect of ceramic thickness on opalescence was not significant (*p* = .211). The interaction effect of ceramic type and ceramic thickness on opalescence was also significant (*p* = .000) with 95% confidence interval. Thus, the factors could not be analyzed independently using post hoc tests. Instead, one‐way ANOVA was applied. Level of significance was set at .05.

**TABLE 2:** Effect of ceramic type and thickness on opalescence

Source	Type II sum of squares	*df*	Mean square	F	Sig.
Corrected model	100.723^a^	15	6.715	22.306	.000
Intercept	399.986	1	399.986	1,328.681	.000
Ceramic type	46.145	3	15.382	51.095	.000
Thickness	0.479	1	0.479	1.590	.211
Ceramic type × thickness	44.785	3	14.928	49.590	.000

^a^ *p* < .05.

## 3 RESULTS

Table 3 presents the mean and standard deviation of opalescence of different ceramic types with 0.5 and 1 mm thicknesses. As shown, in all ceramic groups except for IPS e.max, the mean opalescence of 1‐mm‐thick specimens was higher than that of 0. 5‐mm‐thick specimens. However, the mean opalescence of 0. 5‐mm‐thick specimens of IPS e.max was higher than that of 1‐mm‐thick specimens of this ceramic.

**TABLE 3:** Mean and standard deviation of opalescence of different ceramic types with 0.5 and 1 mm thicknesses

Ceramic type	Thickness	Mean	*SE*	95% confidence interval	
				Lower bound	Upper bound
IPS e.max	0.5 mm	3.398	0.158	3.083	3.713
	1.0 mm	1.470	0.158	1.155	1.785
Enamic	0.5 mm	1.443	0.158	1.128	1.758
	1.0 mm	2.004	0.158	1.689	2.319
Feldspathic	0.5 mm	1.062	0.158	0.747	1.377
	1.0 mm	1.121	0.158	0.806	1.436
Zirconia	0.5 mm	1.980	0.158	1.664	2.295
	1.0 mm	3.853	0.158	3.537	4.168

Table 4 presents the pairwise comparisons of the groups regarding opalescence. As shown, in specimens with 0.5 mm thickness, the opalescence of IPS e.max ceramics was significantly higher than that of feldspathic, zirconia and Enamic ceramics (*p* = .0001). Also, the opalescence of feldspathic ceramic was significantly lower than that of zirconia ceramic (*p* = .001).

**TABLE 4:** Pairwise comparisons of opalescence of different ceramic types with 0.5 and 1 mm thicknesses

Thickness	(I) Ceramic type	(J) Ceramic type	Mean difference (I‐J)	*SE*	Sig.	95% confidence interval for difference	
						Lower bound	Upper bound
0.5 mm	IPS e.max	Enamic	1.955*	0.224	.000	1.349	2.561
		Feldspathic	2.336*	0.224	.000	1.730	2.942
		Zirconia	1.418*	0.224	.000	0.812	2.024
	IPS e.max	Feldspathic	0.382	0.224	.554	−0.224	0.988
		Zirconia	−0.536	0.224	.114	−1.142	0.070
	Feldspathic	Zirconia	−0.918*	0.224	.001	−1.524	−0.312
1.0 mm	IPS e.max	Enamic	−0.534	0.224	.117	−1.140	0.072
		Feldspathic	0.349	0.224	.740	−0.257	0.955
		Zirconia	−2.383*	0.224	.000	−2.989	−1.777
	Enamic	Feldspathic	0.883*	0.224	.001	0.277	1.489
		Zirconia	−1.849*	0.224	.000	−2.455	−1.243
	Feldspathic	Zirconia	−2.732*	0.224	.000	−3.338	−2.126

* Significant values <0.05.

In specimens with 1 mm thickness, the opalescence of IPS e.max, feldspathic and Enamic ceramics was significantly lower than that of zirconia ceramic (*p* = .0001), and the opalescence of Enamic ceramic was significantly higher than that of feldspathic ceramic (*p* = .001).

Graph 1 demonstrates the opalescence of different ceramic specimens with 0.5 and 1 mm thicknesses.

## 4 DISCUSSION

The present study revealed a difference in opalescence of different ceramic types, irrespective of the thickness of specimens. This finding was in agreement with that of Della Bona, Nogueira, and Pecho (2014) They reported that the opalescence of ceramics increased by an increase in concentration of some oxides such as ZrO_2_, Y_2_O_3_, SnO_2_ and V_2_O_5_. Shiraishi, Wood, Shinozaki, and van Noort (2011) reported a strong correlation between the concentration of ZrO_2_ and V_2_O_5_ and opalescence. It seems that higher opalescence of zirconia and e.max, compared with Enamic and feldspathic ceramic, is due to the presence of zirconium oxide in the composition of these ceramics and yttrium oxide present in zirconia.

In our study, the mean opalescence of 1‐mm‐thick specimens of all ceramic types (except for IPS e.max) was higher than that of 0.5‐mm‐thick specimens.

Assuming that absence of translucency is only due to the presence of particles that play a role in opalescence (in other words, the higher the scattering, the lower the translucency), higher opacity of a material would be translated to presence of higher amounts of opalescent materials in its composition. Thus, in equal thickness, we expect the opaquer specimens to have higher opalescence. However, the opalescence of IPS e.max specimens was higher than that of zirconia and Enamic specimens with 0.5 mm thickness. On the other hand, zirconia and Enamic specimens were opaquer than IPS e.max specimens. Thus, this hypothesis was rejected, indicating that aside from the opalescent material, some other factors play a role in reduction of translucency and opacity of specimens.

A ceramic restoration is composed of an opalescent material, ceramic, A2 shade and a masking agent. The lower the amount of the masking agent, the higher the share of the opalescent agent in scattering of blue light would be. Thus, objects with lower masking effect are expected to have higher opalescence, given the optimal grading and volume of opalescent particles. In our study, IPS e.max specimens were more translucent than zirconia and Enamic specimens; thus, this hypothesis may be correct. Although the feldspathic ceramic was more translucent than the zirconia and Enamic ceramics, it should be noted that it does not contain adequate amount of opalescent material; thus, lower masking effect does not apply to this ceramic.

In zirconia and Enamic specimens (but not in IPS e.max), opalescence increased by an increase in thickness. The reason is due to the fact that in higher thicknesses, the light is allowed to transmit through the media since the masking is not complete. Thus, the opalescence is expected to increase. This process is reversed when a 1‐mm‐thick specimen has complete masking. In other words, in complete masking, light does not reach the opalescent material to show opalescence.

Visual assessment of the specimens and their comparison revealed a difference in the masking effect of IPS e.max ceramic in 0.5 and 1 mm thicknesses as it is common for all translucent material according to the well‐known Kubelka‐Munk theory (Diebold, 2014; Kang, 2006). However, the difference in the masking effect of 0.5 and 1 mm thicknesses of zirconia ceramic was insignificant. Thus, the opalescence significantly decreases as the result of increased thickness in IPS e.max specimens due to the severe masking effect. However, in zirconia and Enamic ceramics, change in thickness did not significantly change their masking effect. As a result, light transmission and consequently the opalescence increased. In other words, insignificant change in the masking effect increases the share of opalescent agent. This can be better understood from the transmission curve of IPS e.max and zirconia ceramics in 0.5 and 1 mm thicknesses. A significant reduction was noted in transmission of IPS e.max ceramic by changing the thickness from 0.5 to 1 mm. Thus, the opalescence is expected to decrease. However, in zirconia ceramic, the reduction in transmission was smaller by changing the thickness from 0.5 to 1 mm. Thus, the share of opalescent agent in creation of opalescence increases.

A noteworthy issue in application of ceramics for laminate veneers is that in some cases, restorations fabricated by two different laboratories with A2 shade seem to have different color shades when tried‐in, and one may seem yellower than the other. This can be due to the absence of opalescence. Presence of opalescence in the yellower ceramic would create a blue scattering, conferring a whiter appearance to the ceramic restorations. This issue is more intensified under dental unit light because the unit light is yellow and opalescence plays a major role in this respect.

Armito et al. (2010) compared three different composite types and concluded that the opalescence increased by an increase in thickness. They added that in thicknesses >1 mm, opalescence was influenced by translucency, and translucency significantly decreased by a significant increase in opalescence. In our study, translucency was not the only factor affecting the opalescence, and the amount of opalescent material and the masking effect of specimen also played a role in this respect.

In this regard, it seems important for clinicians to know about opalescence differences in a variety range of ceramic thickness when performing different restoration with different thickness like laminate or crown. Also, it can be helpful for manufacture to become aware of how ceramic thickness can affect optical properties like opalescence and for making more similar restoration to the tooth, may need to change some parameters in ceramics for different purposes.

## 5 CONCLUSION

Within the limitations of this study, it seems that the dental ceramic type and thickness affect the opalescence. In all ceramics evaluated in this study except for IPS e.max, increase in thickness of specimens increased the opalescence. All opalescence values were lower than that of human enamel. Thus, attempts should be continued to find a dental material with an opalescence value similar to that of natural tooth.

### CLINICAL SIGNIFICANCE

Opalescence varies by different types and thickness of dental ceramics. The opalescence values of ceramics are different from tooth enamel. Therefore, manufactures should develop all‐ceramic materials that can simulate the opalescence of natural teeth especially in aesthetic ceramic restoration with lower thickness.
